# Determinant Factors and Characteristics of Injuries Among Preschool Children in Public Daycare Centers

**DOI:** 10.3390/children12020251

**Published:** 2025-02-19

**Authors:** Olga Kouli, Eleftheria Morela, Elissavet Papanikolaou, Antonis Dalakis, Maria Karageorgopoulou, Antonis Kambas

**Affiliations:** Department of Physical Education and Sport Science, Democritus University of Thrace, University Campus, 69100 Komotini, Greece; okouli@phyed.duth.gr (O.K.); emorela@phyed.duth.gr (E.M.); eli.papanikolaou@gmail.com (E.P.); antonydalakis@gmail.com (A.D.); mkarageo@phyed.duth.gr (M.K.)

**Keywords:** unintentional injuries, incidents, preschool center, toddlers, incident report

## Abstract

Background/Objective: Evidence suggests that preschool children experience significantly higher rates of injury-related mortality and disability compared to older age groups of children. However, there seems to be a lack of recorded data on unintentional injuries among preschoolers in public daycare centers in Greece. Therefore, the present study aims to identify determinant factors and characteristics of unintentional injuries among preschool children in public daycare centers. Methods: The sample consisted of 6 daycare centers in the Municipality of Xanthi in Greece, with 444 preschool children (M = 3.73 ± 0.47 years of age). Educators completed reports detailing the number and characteristics of unintentional injuries sustained and provided specific information about each. During the study (10 months), 351 injury reports were completed. Results: Frequency analyses showed that children who were most frequently injured were between 3.5 and 4 years old, while boys were more susceptible. The leading causes of injuries were falls and acute overload. Cuts and wounds were the most common type of injury, and the body part that was more frequently injured was the head. The majority of injuries occurred in classrooms during free play. Conclusions: The results highlight the need for injury prevention strategies in daycare centers, including modifications to play areas, increased supervision during free play, and educator training on risk reduction.

## 1. Introduction

In the 21st century, unintentional injuries are the leading cause of mortality and disability among children and adolescents [1], resulting in 60 to 65% of preventable deaths among children under nine years of age [2]. Notably, injuries between children aged 1–5 years were found to result in the most long-term consequences and are considered to be the primary cause of death among children at this age [3,4]. Furthermore, numerous children worldwide need medical treatment and hospitalization due to injuries and incidents, thus imposing a heavy economic burden on the healthcare system and society [5]. Therefore, it is crucial to determine the features of injuries among preschool-aged children to provide prevention strategies.

Injuries can be caused by exposure to uncontrolled release of physical energy such as heat, electricity, or gravity or by the sudden absence of oxygen [6]. Road traffic injuries, burns, drowning, poisoning, falls, asphyxia, and injuries related to sports are examples of injuries that are not caused on purpose or with harmful intent [7]. Children are generally an age group particularly prone to injuries mainly due to their physical, psychological, and other personality traits and developmental characteristics [8]. Despite the prevalent view that injuries and incidents occur more frequently in preschool years [9], other studies report that the frequency of injuries increases with age [10]. A recent study by Li and colleagues [1] examined injury trends among people aged 0–19 during a three-decade period. Their findings demonstrated, among others, that adolescents aged 15–19 years had higher incidence rates, while children under the age of 5 face a disproportionately higher burden of injury-related mortality and disability compared to older age groups. Similarly, Pearson and Stone [11] reported that infants under 1 year had the highest injury mortality rate (7.1 per 100.000) in Scotland. In Greece, the highest injury mortality rates are found in ages 0–4, while the lowest are in ages 5–9 [12]. This discrepancy in findings across the literature could be attributed to differences in the population studies, data collection methods, types of injuries examined, and contextual influences. Given these differences, country-specific studies are necessary to map the characteristics of unintentional injuries within each population and develop targeted prevention measures.

Previous epidemiologic research at national and international levels has highlighted falls as the leading cause of non-fatal injury in children, followed by being struck by or against an object [13]. Factors that contribute to mortality and morbidity due to injuries are age, gender, socioeconomic status, and ethnicity, to name but a few [14]. Research in the field indicates that boys and preschoolers sustain more unintentional injuries [1,14], while low socioeconomic status is related to an increased risk of injury morbidity [14,15]. In addition, the employment status of parents has been found to influence the risk of injuries, potentially due to factors like time availability for supervision [14]. These factors highlight the importance of awareness, supervision, and preventive measures to reduce the risk of unintentional injuries in children.

Several studies indicated that children under 6 years old are the most vulnerable to injuries [9,16,17], mostly due to their developmental, physical, and behavioral traits [17,18]. Physical characteristics, such as lower body mass, narrower airways, and a more fragile skull structure, contribute to the increased likelihood of unintentional injuries among preschoolers [17,18]. Regarding developmental and behavioral characteristics, preschoolers exhibit high activity levels and a natural curiosity to explore their environment, often making them vulnerable to injuries, especially when supervision is inadequate [19]. In addition, their cognitive limitations and emotional impulsiveness make them struggle to understand imminent dangers and cope effectively with dangerous situations [20]. Despite the unique risk factors associated with this age group, many studies do not distinguish between preschool-aged children and older children [21]. The limited age-specific research makes it challenging to tailor interventions for younger children. Therefore, targeted research on injury patterns among preschoolers is needed to prevent unintentional injuries effectively. Additionally, country-specific research is crucial to understanding local contexts and creating customized prevention strategies.

Like many European countries, Greece faces challenges in preventing unintentional injuries among preschool-aged children. A study focused on injuries occurring in Greek preschool and elementary school settings found that falls were the most common type of unintentional injury, with schoolyards being the primary location [22]. In addition, injuries have also been recorded in Greek camps [23], kindergartens and primary schools [24], playgrounds [25], and sports high schools [26]. However, there is a lack of data on mapping unintentional injuries during early childhood in Greece over the last decades [27]. In addition, there appears to be a lack of recorded data regarding injuries among preschool children in public daycare centers. Therefore, the purpose of the present research was to identify and record the characteristics of injuries occurring in public daycare centers for preschool children, thereby generating data that will contribute to the timely prevention and response to such incidents.

## 2. Materials and Methods

### 2.1. Procedures and Participants

The present study was approved by the Institution’s Ethics Committee, while permission was granted by the local education authorities. The region of Eastern Macedonia and Thrace was initially chosen due to the researchers’ accessibility. Then, a lottery was held among the municipalities within the region, from which the municipality of Xanthi was randomly selected. All the daycare centers in this specific county participated in the research. Before the data collection, the areas of the daycare centers in the municipality of Xanthi, Greece, were mapped, and the daycare managers were contacted by the researchers, who explained the purpose of the study and asked to participate. The researchers provide the daycare managers with injury recording forms to distribute to the educators. The researchers specified that for each accident that occurs, the educators should fill out an accident registration form without any personal details of the children, respecting the children’s personality and following the General Data Protection Regulation (GDPR). The educators were trained by the researchers to complete the accident recording form and provide explanations if required.

During the data collection, all the ethical recommendations for early childhood research developed by Bertram and colleagues [28] were followed. Parental consent was obtained, while preschool children were also informed about the research in age-appropriate language and given the opportunity to provide consent. Participants were informed that they were not obliged to participate and could withdraw consent at any time if needed. In addition, the information recorded by the educators was anonymous and without personal details of the injured children. The researchers ensured that only necessary information should be collected, and the data will be used exclusively for the present research. Throughout the research process, the researchers did not contact the children in any way or enter their space. The data collection took place at regular intervals by the researchers during an academic year and after appointments with the daycare managers. All daycare centers (N = 6) took part in the study, with a total number of participants of 444 children (188 girls) and 23 educators. The children’s ages ranged from 3 to 5 years. Educators completed 351 injury reports gathered over a school year.

### 2.2. Instrument

Educators completed the standardized accident registration form “Student Injury and Incident Report for use in Swedish Schools” [29], modified for the Greek population [30]. This form has been validated by its original developers, Laflamme and colleagues [31], and further validated for the Greek population by Papageorgiou and colleagues [30] through the use of Factorial Analysis of Correspondence (FAC) and Hierarchical Ascendant Classification (HAC) in the respective studies. These analytical methods play a crucial role in enhancing the validity of research findings and complement each other in exploring data, uncovering underlying structures, and assessing result consistency, thereby strengthening the robustness of the conclusions drawn from the data [32,33]. The form consists of 14 categories of questions that aim to identify the characteristics of the incidents and injuries related to (i) the age and the gender of the children who were injured, (ii) the location where the injury took place (indoor and outdoor location), (iii) the activity during which the injury occurred, (iv) the part of the body that was injured, and (v) the type of injury that was caused.

### 2.3. Data Analysis

Frequency analyses were conducted to determine the frequency, causes, and location of unintentional injuries among preschool children, as well as the frequency distribution for children’s gender and age group.

## 3. Results

### 3.1. Unintentional Injuries by Age and Gender

Descriptive statistics of the participants are presented in Table 1. A total of 351 unintentional injuries were reported over the school year. Of the injured children (N = 351), 63.8% were boys and 36.2 were girls. The children who were most frequently injured were between 4 years old (39%) and 3.5 years old (32.5%).

### 3.2. Activity and Location of Injury Occurrence

**Figure 1:**
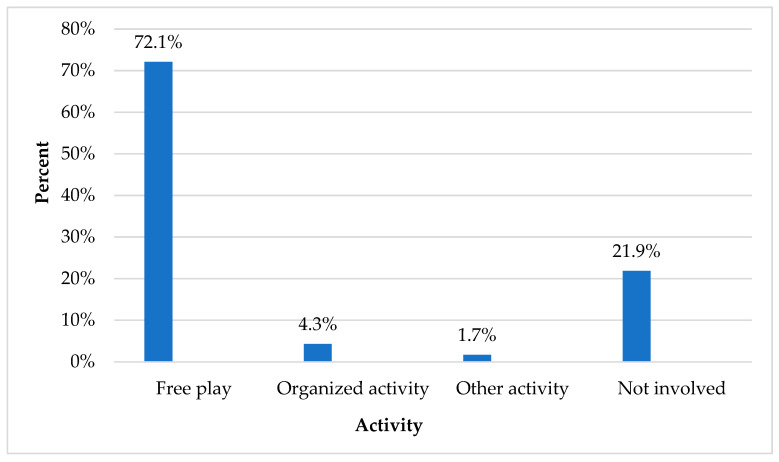
Activity in which the injury occurred.

Most children were injured in free play (72.1%) followed by organized activity (4.3%) (Figure 1).

### 3.3. Location of the Injuries (Indoors and Outdoors)

**Figure 2:**
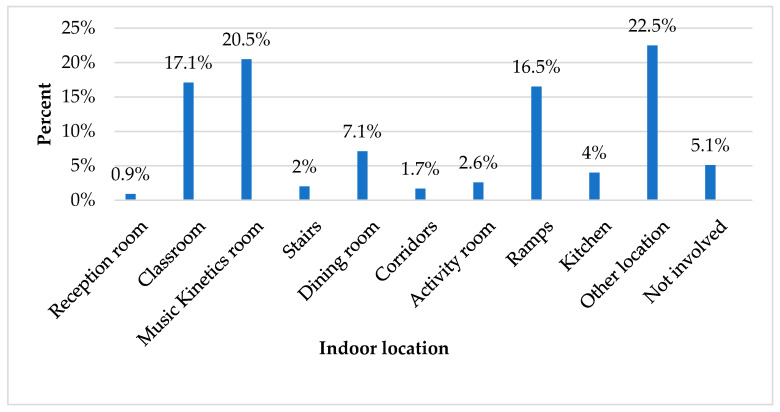
Indoor location.

**Figure 3:**
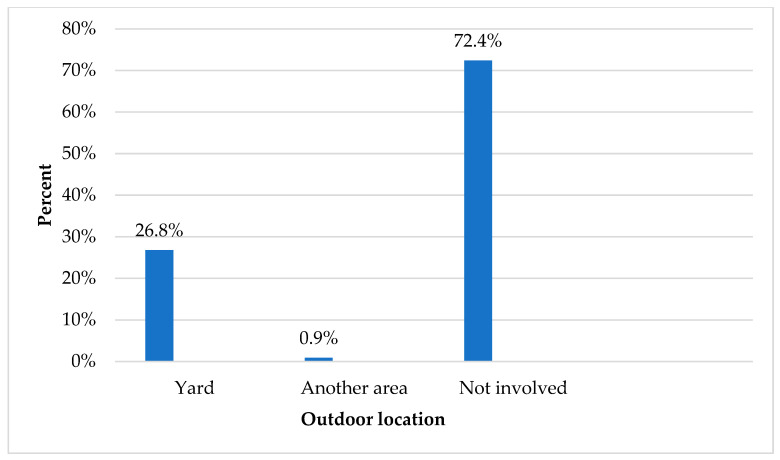
Outdoor location.

Regarding the indoor location, the music kinetics room (20.5%), the classroom (17.1%), and the ramps (16.5%) were the most commonly reported (Figure 2). Regarding outdoor location, the frequency of injuries occurred in the yard (26.8%), while, interestingly, the vast majority of students were not injured in outdoor locations (72.4%) (Figure 3).

### 3.4. Injury Characteristics

**Figure 4:**
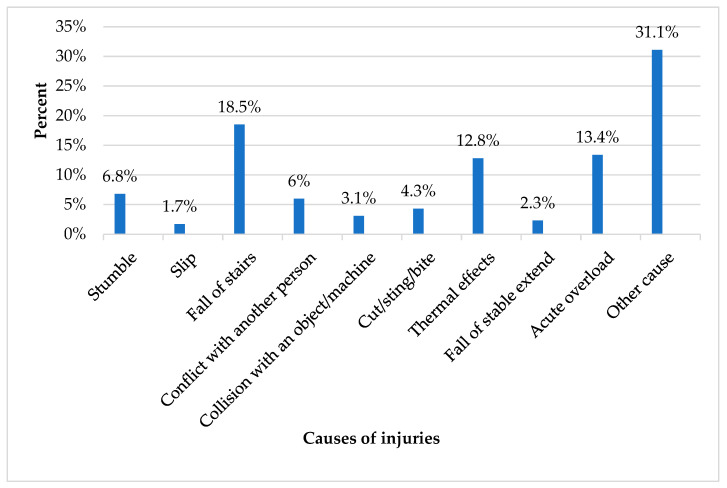
Causes of injuries.

**Figure 5:**
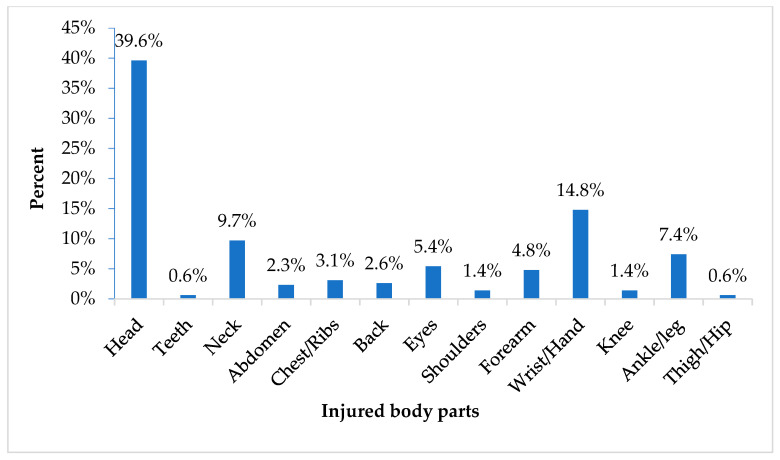
Injury by body part.

**Figure 6:**
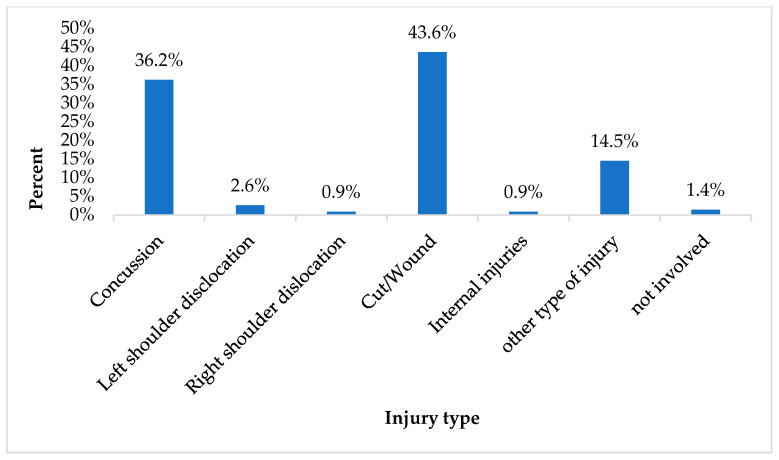
Injury by type.

The leading cause of injuries was falling down the stairs (18.5%), followed by acute overload, which refers to injuries caused by sudden and excessive physical demands that exceed an individual’s immediate capacity to handle (13.4%) (Figure 4). The most frequently injured body part was the head (39.6%), followed by the wrist/hand (14.8%) (Figure 5), while the most common injury type was cut/wound (43.6%) followed by concussion (36.2%) (Figure 6).

## 4. Discussion

To the best of our knowledge, this is the first study to describe unintentional injuries among preschool children attending public daycare centers in Greece. The findings revealed that the highest percentage of injuries in the areas of the daycare centers occurred among children aged 3.5 to 4 years old. The present finding corresponds with a large part of the literature that considers preschoolers the most vulnerable age group for injuries [9,17]. Our findings suggest that children within this age range are particularly vulnerable to unintentional injuries, likely due to their increased tendency to explore their environment while lacking a comprehensive understanding of potential dangers. In addition, the results indicated that boys were more likely to experience an injury than girls, a trend that is consistent with the majority of studies in the field documenting a higher risk of injury among boys compared to girls [34].

In addition, regarding the kind of activity and the location of the injuries, the results revealed that most of the injuries occurred during free play in the teaching areas. Previous studies in the field found that many school injuries happen during playtime and in classrooms for all school-aged children [35]. This is probably because of the sense of freedom that children experience, the absence of specific rules in such activities, and the inadequate supervision during playtime that may increase the risk of injury. Furthermore, regarding injury characteristics, the results revealed that the leading cause of injuries was falling, while the most frequently injured body part was the head. These findings coincide with the existing literature highlighting that falls are a common cause of fatal and serious head injuries in young children [5,36]. Preschool children, especially those who are still developing important motor skills like balance and coordination, are therefore more prone to falls. Furthermore, the most commonly reported injuries in our study were cuts and wounds, which is consistent with findings reported by other studies [37].

Overall, the present findings offer valuable information regarding the frequency, causes, and places of unintentional injuries among preschool children, thus filling the gap in the literature on early childhood injury. The relatively large sample (444 children) and the extended study duration (10 months) enhance the reliability of the findings and offer practical insights that can inform targeted injury prevention strategies in daycare centers. Despite the importance of the present results, the descriptive design of our study cannot establish cause-effect relationships. The study was conducted in a specific area in Northern Greece, so the results cannot be extrapolated to the whole population. More localized research is needed to understand the dynamics of injuries occurring within daycare centers and to develop target prevention strategies. Furthermore, the present study did not provide enough information about important determinants of unintentional injuries, such as the adequacy of daycare centers’ facilities, educators’ supervision, and other important child factors such as physical and mental health conditions. For future research, it is important to consider more risk factors associated with unintentional injuries to design effective prevention programs. Lastly, the data are prone to reporting bias by the survey respondents (children’s educators) since they record the unintentional injuries of the children; therefore, we must be cautious when interpreting the data. Observational studies or parental reports could complement these findings to enhance accuracy.

## 5. Conclusions

Unintentional injuries in school contexts are common worldwide, including in preschool settings, and are a significant concern for child safety. Early intervention, awareness, and proactive safety measures can greatly reduce the occurrence and severity of these injuries. In Greece, there is limited research focusing on injuries in school settings and a notable gap in data on injuries that occur in daycare centers. The present study offers valuable insights into the determinants and characteristics of unintentional injuries in preschool children in Greece, highlighting the need for proactive injury prevention in daycare centers. Prevention strategies may include redesigning play areas to minimize fall risks, providing rest periods, enhancing supervision during free play, and providing educators with specialized training in injury prevention. The results offer valuable guidance for local decision-makers, enabling them to design specific strategies to prevent injuries in preschool settings in the area, thus fostering safer environments for preschoolers.

## Figures and Tables

**Table 1:** Sports injuries by age and gender.

Sports Injuries by	Participants (N)	Relative Values (%)
Age		
3 yrs	N = 60	17.1%
3.5 yrs	N = 114	32.5%
4 yrs	N = 137	39%
4.5 yrs	N = 35	10%
5 yrs	N = 5	1.4%
Total	N = 351	
Gender		
Boys	N = 224	63.8%
Girls	N = 127	36.2%
Total	N = 351	

## Data Availability

The data that support the findings of this study are available from the first author upon reasonable request.
